# Persimmon-derived tannin has bacteriostatic and anti-inflammatory activity in a murine model of *Mycobacterium avium* complex (MAC) disease

**DOI:** 10.1371/journal.pone.0183489

**Published:** 2017-08-21

**Authors:** Yoko Matsumura, Masahiro Kitabatake, Noriko Ouji-Sageshima, Satsuki Yasui, Naoko Mochida, Ryuichi Nakano, Kei Kasahara, Koichi Tomoda, Hisakazu Yano, Shin-ichi Kayano, Toshihiro Ito

**Affiliations:** 1 Department of Immunology, Nara Medical University, Kashihara, Nara, Japan; 2 Department of Health and Nutrition, Faculty of Health Science, Kio University, Kitakatsuragi-gun, Nara, Japan; 3 Department of Microbiology and Infectious Diseases, Nara Medical University, Kashihara, Nara, Japan; 4 Center for Infectious Diseases, Nara Medical University, Kashihara, Nara, Japan; 5 Second Department of Internal Medicine, Nara Medical University, Kashihara, Nara, Japan; University of British Columbia, CANADA

## Abstract

Nontuberculous mycobacteria (NTM), including *Mycobacterium avium* complex (MAC), cause opportunistic chronic pulmonary infections. Notably, MAC susceptibility is regulated by various factors, including the host immune system. Persimmon (Ebenaceae *Diospyros kaki* Thunb.) tannin is a condensed tannin composed of a polymer of catechin groups. It is well known that condensed tannins have high antioxidant activity and bacteriostatic properties. However, it is hypothesized that condensed tannins might need to be digested and/or fermented into smaller molecules *in vivo* prior to being absorbed into the body to perform beneficial functions. In this study, we evaluated the effects of soluble persimmon-derived tannins on opportunistic MAC disease. Soluble tannins were hydrolyzed and evaluated by the oxygen radical absorbance capacity (ORAC) method. The ORAC value of soluble tannin hydrolysate was approximately five times greater than that of soluble tannin powder. In addition, soluble tannin hydrolysate exhibited high bacteriostatic activity against MAC *in vitro*. Furthermore, in an *in vivo* study, MAC infected mice fed a soluble tannin-containing diet showed significantly higher anti-bacterial activity against MAC and less pulmonary granuloma formation compared with those fed a control diet. Tumor necrosis factor α and inducible nitric oxide synthase levels were significantly lower in lungs of the soluble tannin diet group compared with the control diet group. Moreover, proinflammatory cytokines induced by MAC stimulation of bone marrow-derived macrophages were significantly decreased by addition of soluble tannin hydrolysate. These data suggest that soluble tannin from persimmons might attenuate the pathogenesis of pulmonary NTM infection.

## Introduction

Nontuberculous mycobacteria (NTM) are organisms present in water, soil, and throughout our environment [1,2]. NTM, including *Mycobacterium avium* complex (MAC), causes chronic pulmonary infection, especially in immune-deficient people, however, the factors involved in MAC pathogenesis are not identified. These organisms are more resistant to antibiotics than *Mycobacterium tuberculosis*, and an effective treatment has yet to be established. MAC is the most common NTM that causes disease in humans [3]. As NTM disease is an opportunistic infection, there may be a positive association between this disease and host factors.

A number of studies have demonstrated that MAC disease is associated with the host immune system [4–7]. A granuloma is a characteristic pathophysiological feature induced by mycobacterial infections, and is an important element of the host defense system. The typical mycobacterial granuloma contains macrophages and dendritic cells surrounded by T lymphocytes, with macrophages being the dominant cell type found in granulomas [8]. During mycobacterial infection, macrophages become activated and produce proinflammatory cytokines, including interleukin (IL)-12 and tumor necrosis factor (TNF)-α, and nitric oxide (NO) to protect against mycobacterial infection and create a T-helper (Th) 1-inducing microenvironment. It is well known that macrophages can be activated by either a classical (M1) or alternative (M2) pathway depending on the stimulus. M1 macrophages, induced by the Th1-dependent cytokine interferon (IFN)-γ, play an important role in the elimination of various pathogens including MAC [8]. They upregulate the expression of proinflammatory cytokines (IL-1β, IL-6, and TNF-α) and via activation of inducible NO synthase (iNOS) are able to secrete NO, a free radical that is toxic to bacteria and other pathogens. Th1 cells also play a central role in protection against intracellular pathogens and induce activation of macrophages. Thus, they are deeply involved in inflammation and granuloma formation, which play critical roles in protection against MAC [4].

Persimmon (Ebenaceae *Diospyros kaki* Thunb.) tannin is a condensed tannin composed of a polymer of flavan-3-ols including catechin groups. The astringent persimmon fruits contain large quantities of tannin, which is polymer of catechin group such as epigallocatechin gallate, epigallocatechin, epicatechin gallate and epicatechin. These four catechins are mainly condensed and formed large molecule ranging from 7 to 20 kDa (degree of polymerization 19–47) [9], and show high antioxidant activity [10–14]. Dietary antioxidants may reduce oxidative stress to DNA, proteins and lipids; however, it is important that these antioxidants act as efficiently *in vivo* as *in vitro*. We have demonstrated that the plasma oxygen radical absorbance capacity (ORAC) value of rats fed the non-extractable fraction of persimmon fruit, which contains condensed tannin, was higher than the control diet group. This suggests that condensed tannin may be fermented by enteric bacteria, followed by absorption as smaller antioxidant molecules *in vivo* [15].

De la Fuente et al. showed that antioxidants have immunomodulatory functions [16]. Notably, hydrolyzed tannins inhibited NO production by the murine macrophage cell line RAW264.7 activated with lipopolysaccharide and IFN-γ *in vitro* [17].

In this article, we focused on the antioxidant activity of persimmon-derived condensed tannin and MAC disease, and evaluated whether the tannin has any immunological benefits in MAC disease. If the tannin is effective against MAC disease, it may be a valuable new therapeutic lead against opportunistic infection by MAC.

## Materials and methods

### Tannin preparation

We used fruits of astringent persimmon (Ebenaceae *Diospyros kaki* Thunb., cv. ‘Tone-wase’ and ‘Hiratane-nashi’) for preparation of soluble tannin. ‘Hiratane-nashi’ was designated as a protected species No.24, Niigata Prefecture, Japan in 1962. ‘Tone-wase’ is mutated variety of ‘Hiratane-nashi’, and was registered for ‘The Plant Variety Protection No.28’, Ministry of Agriculture, Forestry and Fisheries, Japan in 1980. In this study, the species of persimmon were identified by Sadahiro Hamasaki, Chief Researcher of Nara Prefecture Agricultural Research and Development Center, scientist No.80393396 of electric research and development (e-Rad) in Japan, and soluble tannin was prepared from immature fruits of these persimmons according to the modified method in his study [18]. Briefly, immature persimmon fruit, which were harvested in Gojo City, Nara, Japan in 2011, were treated with 0.2% (v/w) ethanol for 5 days to insolubilize tannin. The treated fruits were crushed into small pieces, water added, and kept for 2 days at room temperature. The preparation was then divided into supernatant, which contains soluble components such as sugars, and decanted residue which contains insoluble tannin. For extracted tannins, water was added to the residue and heated at 120°C for 30 min to extract insoluble tannin as soluble tannin. The extract was filtrated, evaporated *in vacuo*, and drum dried at 160°C to obtain soluble tannin powder. A batch of soluble tannin powder contained 75.5% condensed tannin, which was measured as epigallocatechin gallate equivalent on the basis of the Folin-Ciocalter method [18]. The soluble tannin powder was prepared and provided by Ishii-Bussan, Inc. (Nara, Japan), and stored at −20°C until use.

### Preparation of soluble tannin hydrolysate

Portions (100 mg) of soluble tannin powder were dissolved in 5 ml of a 1.2 N HCl–50% methanol solution, heated at 90°C for 3 hours, and then diluted to a final volume of 10 ml using the same solution.

### ORAC assay of tannin

ORAC values of soluble tannin powder and soluble tannin hydrolysate were measured according to previously described methods [19–20] with slight modifications. This assay was performed based on the principle that antioxidant compounds delay decreases in fluorescein following the addition of the peroxyl radical generator 2,2-azobis (2-amidinopropane) dihydrochloride (AAPH). ORAC assays were performed using an ARVO™ X4 microplate reader (PerkinElmer, Inc., Waltham, MA, USA) at an excitation wavelength of 485 nm and an emission wavelength of 535 nm.

The fluorescence of each microplate well was measured every 2 min over a 90 min period at 37°C. The area under the fluorescence curve was calculated, and the ORAC values for each sample were expressed as units per 1 μmol equivalents of Trolox (TE). Each sample was measured in triplicate, and data are expressed as means ± standard error of mean (SEM).

### Bacteriostatic activity of soluble tannin hydrolysate against MAC

#### Strains of MAC

MAC strains were isolated from patients at Nara Medical University Hospital (Nara, Japan), and were kindly provided by Dr. Koichi Tomoda (MAC-1), and Dr. Kei Kasahara (MAC-2 and MAC-3), respectively.

#### Bacteriostatic activity against MAC

MAC was grown to mid-log phase in Middlebrook 7H9 liquid medium with glycerol and Middlebrook albumin-dextrose-catalase enrichment (Difco Laboratories, Detroit, MI, USA), aliquoted, and frozen at −80°C until use.

2×10^2^ colony forming units (CFU) of MAC were inoculated into 2 ml Middlebrook 7H9 broth for cultivation. To evaluate dose-dependent actions of soluble tannin, 20 μl soluble tannin in water or soluble tannin hydrolysate in 1.2 N HCl–50% methanol solution were added corresponding to 0, 30, or 100 μg/ml soluble tannin powder per tube. These tubes were incubated at 37°C for 1 week. Bacterial counts were determined by plating serial dilutions of culture liquid onto Middlebrook 7H10 agar plates with oleic acid-albumin-dextrose-catalase enrichment (Difco) and counting bacterial colonies after a further week of culture.

### Animal studies

#### Animals, diet and MAC infection

Seven-week-old female BALB/c mice were purchased from Japan SLC (Hamamatsu, Shizuoka, Japan). Mice of the control diet group were fed an AIN-93G-modified basal diet (CLEA Japan Inc., Tokyo, Japan) and mice of the soluble tannin diet group were fed the basal diet supplemented with 2% soluble tannin powder substituted for an equal amount of cellulose. The composition of study diets are presented in Table 1. One week after study diets were commenced, mice were anesthetized with pentobarbital, and infected by administration of 1×10^7^ CFU MAC (MAC-1) in 50 μl saline with a 26-gauge needle via the trachea. Control mice were treated with 50 μl saline. The animals were fed *ad libitum*, and body weights were monitored every week. At 8 weeks after MAC infection, mice were euthanized by blood collection from left ventricular of heart following pentobarbital anesthesia, and lung samples harvested. The left lobe of the lung was used for histological assessment, and the right lobes were homogenized for the analysis of mRNA and CFU measurement. The experimental protocols were approved by the Ethics Review Committee for Animal Experimentation of Nara Medical University (approval number 11553).

**Table 1:** Compositions of diets (%).

Ingredients	Control diet^*a*^	Soluble tannin diet^*b*^
corn starch casein α-corn starch sucrose soybean oil mineral mix^*c*^ vitamin mix^*d*^ _L_-cysteine choline bitartarate crystalline cellulose soluble tannin powder^*e*^	39.75 20.0 13.2 10.0 7.0 3.5 1.0 0.3 0.25 5.0 -	39.75 20.0 13.2 10.0 7.0 3.5 1.0 0.3 0.25 3.0 2.0

^*a*^ The AIN-93G-modified basal diet without di-*tert*-butylhydroxytoluene was used as the control diet.

^*b*^ Soluble tannin diet comprised of the control diet supplemented with 2% soluble tannin powder substituted for an equal part cellulose (w/w).

^*c*^ Mineral mix AIN-93G.

^*d*^ Vitamin mix AIN-93G.

^*e*^ The soluble tannin powder was prepared and provided by Ishii-Bussan, Inc. (Nara, Japan).

#### Lung histology

Individual excised lung lobes were inflated and fixed with 10% buffered formalin for morphometric analysis. The areas of the granulomas were measured in a blinded fashion on hematoxylin and eosin (H&E)-stained sections of paraffin-embedded lung tissue using computer-assisted morphometry as previously described [8]. A minimum of 10 granulomas per lung section were analyzed to determine average granuloma size.

#### Bacterial counts per lung lobe

Lungs were removed and a lobe of lung was homogenized in 1 ml sterile saline. Bacterial counts per lung lobe were determined by plating serial dilutions of lung homogenates with sterile saline from individual mice onto Middlebrook 7H10 agar plates and counting the number of bacterial colonies.

### Bone marrow-derived macrophage (BMDM) generation and culture

For generation of BMDMs, BM cells were isolated from the tibia and femur of BALB/c mice, and were cultured in L929 cell-conditioned medium as described previously [21–22]. Six days after initial BM cell culture, BMDMs were washed and transferred to 24 well plates using RPMI 1640 medium with L-glutamine and HEPES (Wako Pure Chemical Industries, Osaka, Japan), supplemented with 10% heat-inactivated fetal bovine serum and 1% penicillin/streptomycin solution. After overnight incubation, cells were then treated with soluble tannin hydrolysate for an hour, and then stimulated with MAC (MOI = 50) for 6 hours for RNA analysis. BMDMs treated with 1.2 N HCl–50% methanol solution were used as controls. Cell viability was greater than 93% following exposure to 100 μg/ml soluble tannin hydrolysate and MAC compared with the negative control (untreated) viability, as determined by trypan blue. Cells were collected for analysis of mRNA by quantitative real-time PCR (qPCR), and supernatants were collected for ELISA analysis for cytokine production.

### Quantitative real-time PCR (qPCR)

A part of the removed lung was immersed in 0.5 ml RNAlater® Stabilization Solution (Thermo Fisher Scientific Inc., Waltham, MA) overnight at 4°C, and stored at −80°C until RNA extraction. Total RNA was extracted from the lung using NucleoSpin® RNA (MACHEREY-NAGEL GmbH & Co. KG, Düren, Germany) and stored at −80°C.

Total RNA from BMDMs was extracted using High Pure RNA Isolation Kit (Roche Diagnostics GmbH., Mannheim, Germany) and stored at −80°C.

cDNA from total RNA was prepared using High-Capacity cDNA Reverse Transcription Kit (Applied Biosystems, Waltham, MA). qPCR was performed with Taqman gene expression assays using Step One™ qPCR system (Applied Biosystems). mRNA expression was analyzed by the ∆∆Ct method and normalized to GAPDH expression as previously described [5].

### ELISA analysis of cytokine expression

Murine IL-1ß, IL-6, and TNF-α levels were measured in 50 μl samples from BMDM supernatant or whole lung homogenates using sandwich ELISA kits (Thermo Fisher Scientific Inc.) according to the manufacturer’s instructions. For lung samples, cytokine levels were normalized to the protein concentration (in mg) present in the cell-free preparation of each sample measured by the Bradford assay, as described previously [23].

### Statistical analysis

Statistical significance was evaluated by analysis of variance. *P* values of < 0.05 were considered to indicate statistically significant differences. All statistical analyses were performed using GraphPad Prism 4.0 (GraphPad Software, San Diego, CA). All data are presented as the mean ± SEM and are representative of at least two independent experiments.

## Results

### ORAC value of persimmon-derived tannin

**Fig 1:**
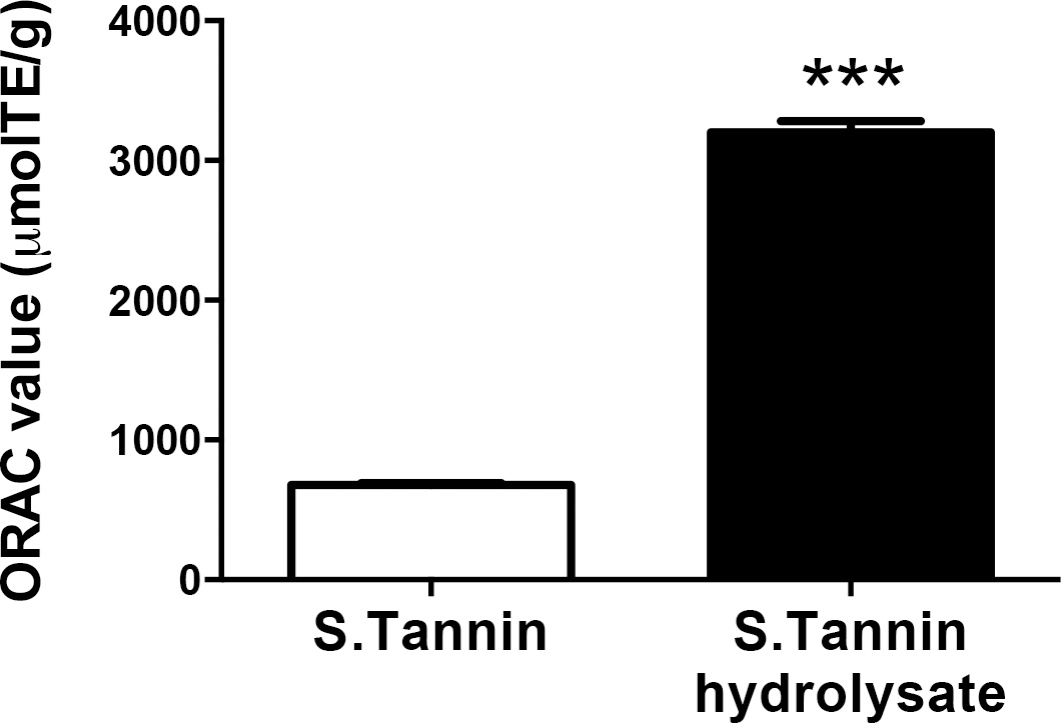
ORAC values of soluble tannin powder and soluble tannin hydrolysate. Soluble tannin powder was hydrolyzed by heating at 90°C for 3 hours with 5 ml of a 1.2 N HCl–50% methanol solution, followed by dilution to 10 ml using 1.2 N HCl–50% methanol solution. The values are presented as means ± SEM (n = 3). *** *P* < 0.001 compared with non-hydrolyzed soluble tannin powder.

We first examined the antioxidant activity of soluble tannin hydrolysate by the ORAC assay method. The ORAC value of soluble tannin hydrolysate was 3202 μmol TE/g, which was approximately five times greater than soluble tannin powder (Fig 1). This suggests that soluble tannin requires hydrolysis to enhance its antioxidant activity.

### Bacteriostatic activity of soluble tannin hydrolysate against MAC

**Fig 2:**
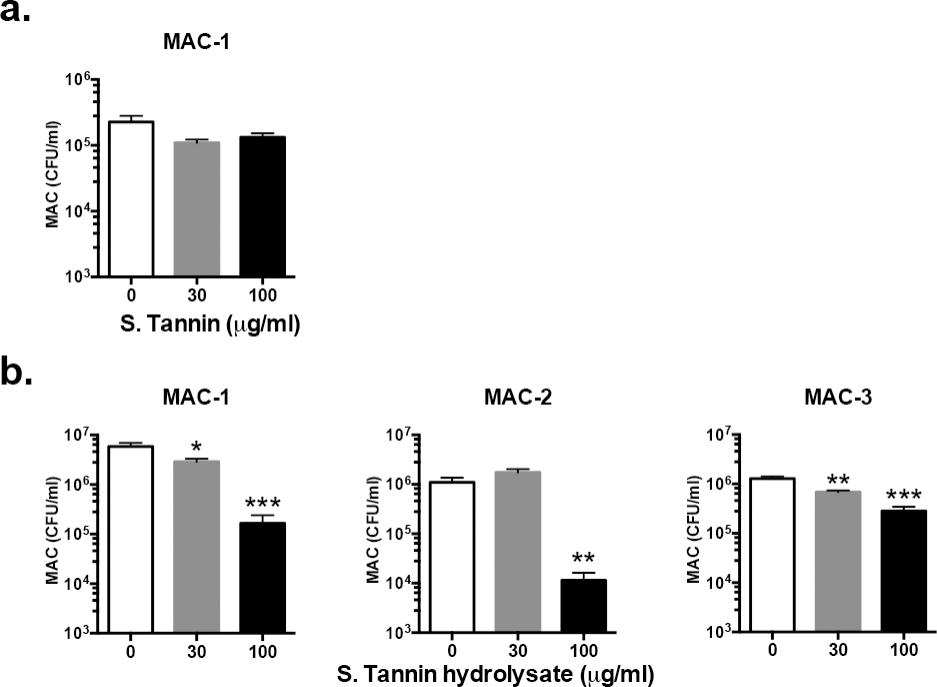
Analysis of soluble tannin and soluble tannin hydrolysate bacteriostatic activity against MAC. MAC strains (2×10^2^ CFU each) were inoculated in culture medium with soluble tannin (hydrolysate). Soluble tannin (a) and soluble tannin hydrolysate (b) was added at 0, 30, and 100 μg/ml soluble tannin powder to each of three tubes and the CFU determined. The values are presented as means ± SEM (n = 4). * *P* < 0.05 when compared with no treatment with soluble tannin hydrolysate (0 μg/ml).

We next examined whether soluble tannin and soluble tannin hydrolysate have direct anti-bacterial activity against MAC. First, we investigated anti-bacterial activity against MAC by soluble tannin, but soluble tannin itself did not inhibit MAC growth (Fig 2A). Then, we next examined anti-bacterial activity against MAC by soluble tannin hydrolysate. As shown in Fig 2B, the CFU of MAC treated with soluble tannin hydrolysate was significantly lower than that without soluble tannin hydrolysate. Furthermore, the anti-bacterial activity against MAC was also seen in other two MAC strains (MAC-2 and MAC-3) examined (Fig 2B). Therefore, soluble tannin hydrolysate was used in further *in vitro* studies.

### Persimmon-derived tannin protects against MAC disease *in vivo*

**Fig 3:**
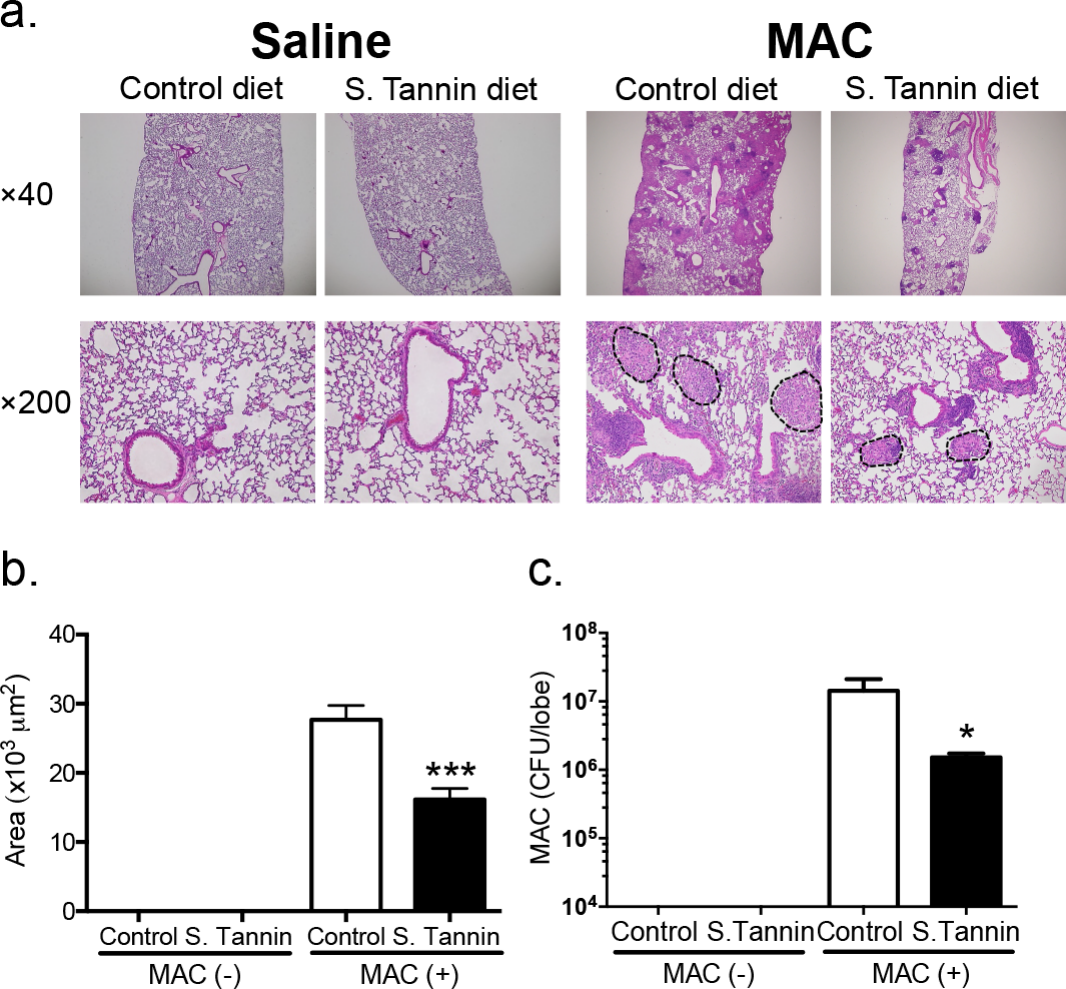
The effect of soluble tannin in a MAC-infected pulmonary granuloma model. Diets were started 1 week before MAC infection. All mice were analyzed at 8 weeks after MAC infection. (a) Microscopic morphology of granulomas in the H&E-stained lungs of MAC-infected BALB/c mice fed control and 2% soluble tannin diets. Dotted lines indicate the border of granulomas. Original magnification, ×40 and ×200. (b) Analysis of the size of lung granulomas between control and 2% soluble tannin diet. (c) MAC CFU counts in one lobe of lungs using Middlebrook 7H10 agar plates as described in the Materials and Methods. The values are presented as means ± SEM (uninfected mice, n = 5; MAC-infected mice, n = 8). * *P* < 0.05, *** *P* < 0.001 compared with MAC-infected mice fed the control diet.

To directly examine anti-bacterial activity of persimmon-derived tannin against MAC *in vivo*, mice were fed a diet containing soluble tannin for 9 weeks from 1 week before MAC infection. The soluble tannin diet group demonstrated formation of significantly smaller granulomas compared with the control diet group (Fig 3A). Quantification and morphometric analysis of granulomatous lesions in lungs is presented in Fig 3B. In mice fed the soluble tannin diet, the size of granulomas was significantly smaller than control diet group (Fig 3B). Moreover, bacterial counts in the lobes of lungs infected with MAC from mice fed the soluble tannin diet were significantly less than those of the control diet group (Fig 3C). These data suggest that persimmon-derived soluble tannin exhibits bacteriostatic activity against MAC both *in vitro* and *in vivo*, and inhibits the granulomatous inflammatory response.

**Fig 4:**
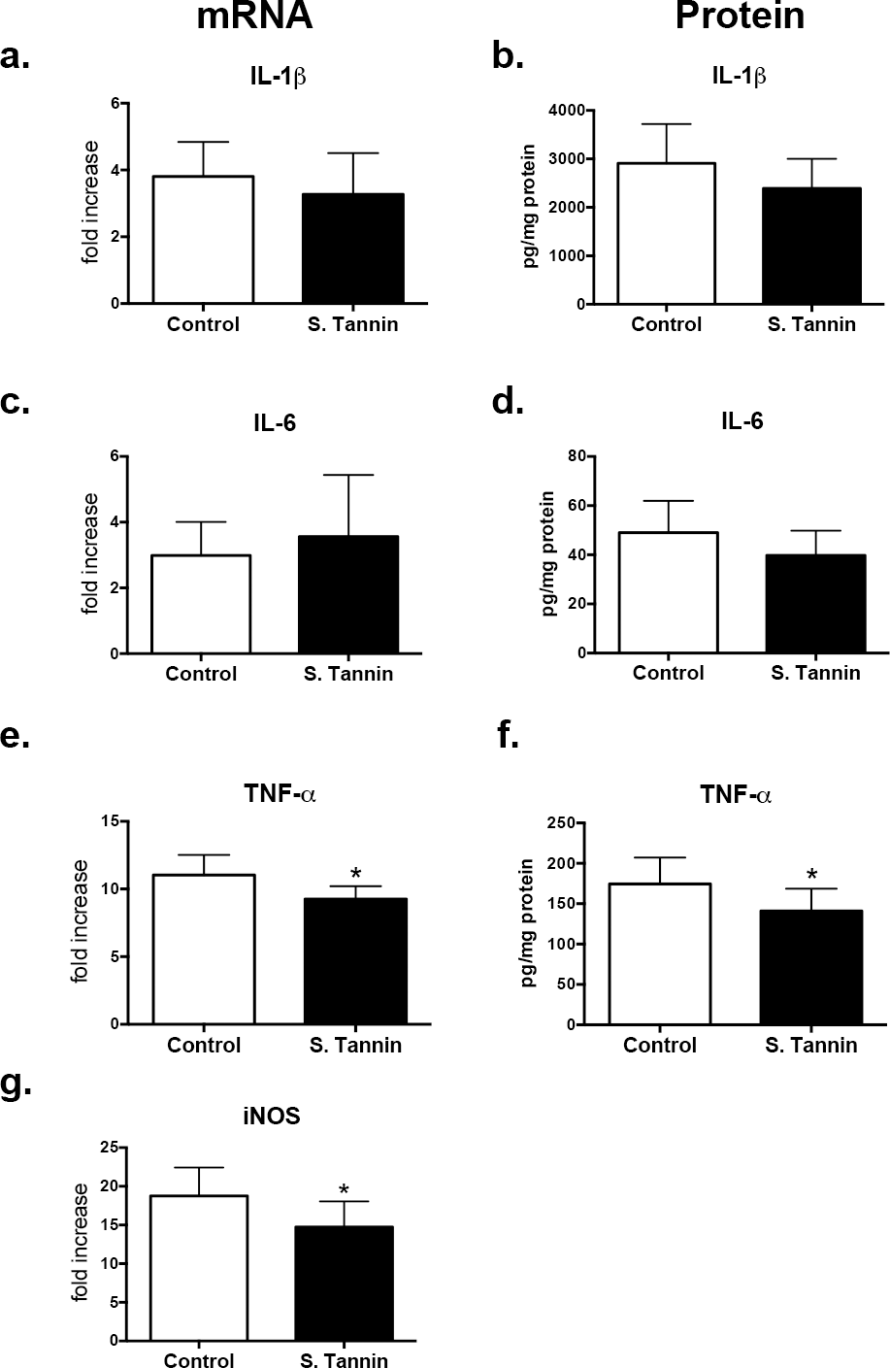
mRNA and protein levels of inflammatory cytokines (IL-1ß, IL-6, TNF-α) and iNOS gene expression of lungs from MAC-infected BALB/c mice fed control and 2% soluble tannin diets. mRNA levels of IL-1ß (a), IL-6 (c), TNF-α (e), and iNOS (g) were quantitated as described in the Methods. Total RNA was extracted from lungs, reverse-transcribed into cDNA and qPCR performed. The protein level of IL-1ß (b), IL-6 (d), and TNF-α (f) was determined in the whole lungs from MAC-infected mice by ELISA as described in the Methods. The values are presented as means ± SEM (uninfected mice, n = 5; MAC-infected mice, n = 8). * *P* < 0.05 when compared with MAC-infected mice fed the control diet.

Next, to help elucidate the mechanism underlying the changes in pulmonary granuloma size and bacteriostatic activity during MAC infection in control and soluble tannin diet, we evaluated the profile of inflammatory genes in the granulomatous lungs. Gene expression and protein levels of TNF-α and iNOS were significantly lower in the soluble tannin diet group compared with the control group, while there was no significant difference in IL-1ß and IL-6 levels between these groups (Fig 4).

### Cytokine levels and iNOS expression by BMDM following MAC infection

**Fig 5:**
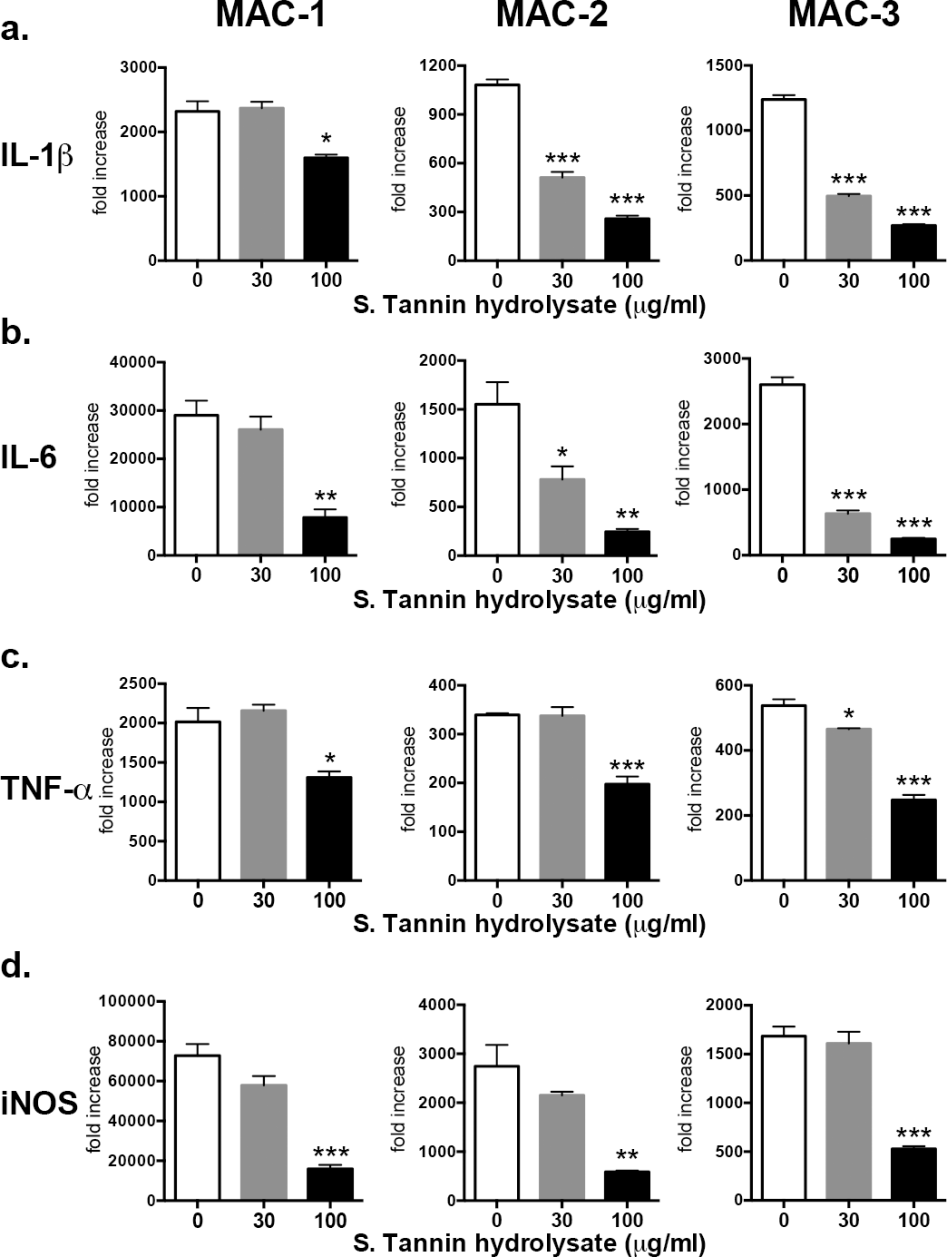
Gene expression of proinflammatory cytokines (IL-1ß, IL-6, TNF-α) and iNOS in BMDMs treated with soluble tannin hydrolysate. mRNA levels of IL-1ß (a), IL-6 (b), TNF-α (c), and iNOS (d) were determined by real-time PCR. BMDMs were pre-treated with soluble tannin hydrolysate (0, 30, and 100 μg/ml) for 1 hour followed by stimulation with three different strains of MAC (MAC-1, MAC-2, and MAC-3) for 6 hours. mRNA was extracted from BMDMs, reverse-transcribed into cDNA and qPCR performed. The values are presented as means ± SEM (n = 3–4). * *P* < 0.05, ** *P* < 0.01, *** *P* < 0.001 compared with no treatment with soluble tannin hydrolysate (0 μg/ml).

**Fig 6:**
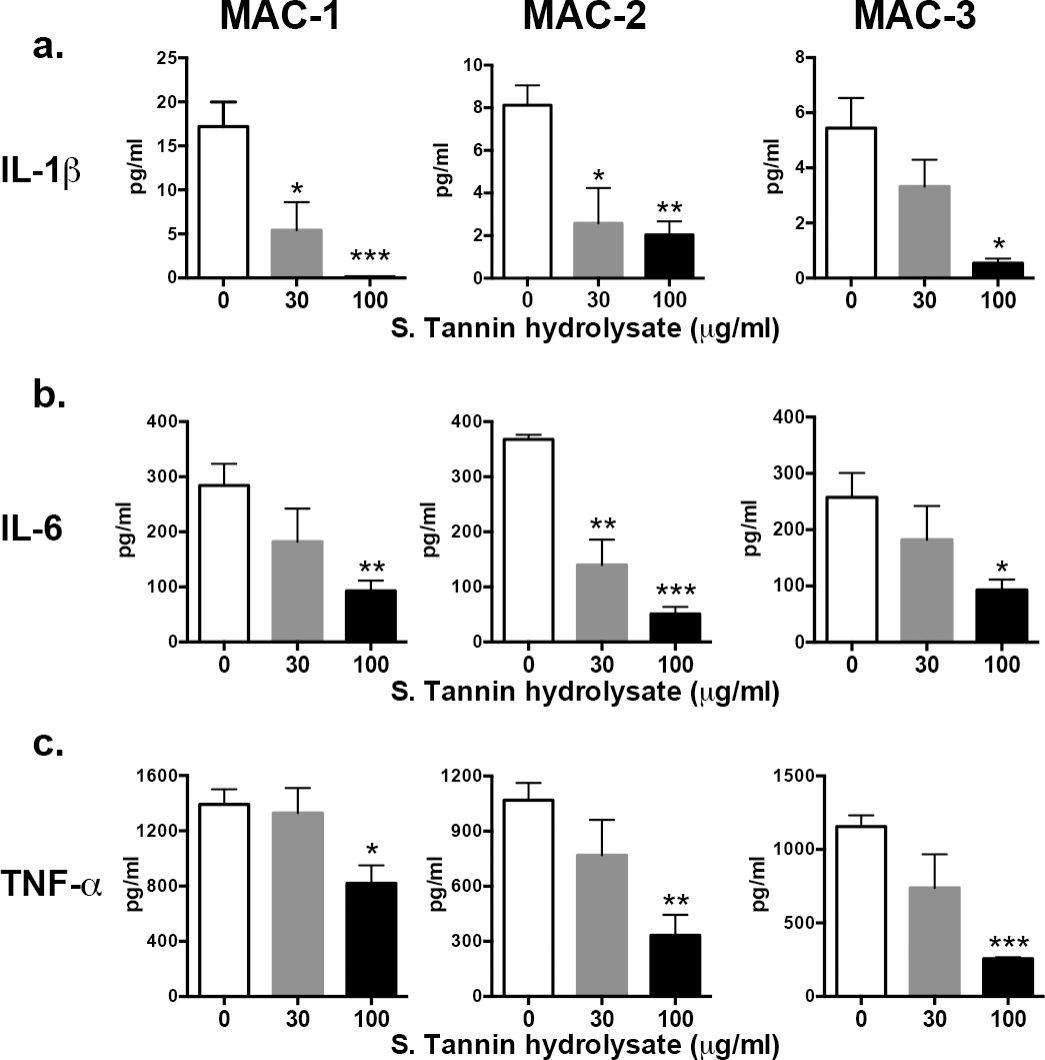
Proinflammatory cytokine (IL-1ß, IL-6, TNF-α) secretion by BMDMs treated with soluble tannin hydrolysate. BMDMs were pre-treated with soluble tannin hydrolysate (0, 30, and 100 μg/ml) for 1 hour followed by stimulation with three different strains of MAC (MAC-1, MAC-2, and MAC-3) for 6 hours. The supernatant was harvested and IL-1ß (a), IL-6 (b), and TNF-α (c) protein concentration was determined by ELISA. The values are presented as means ± SEM (n = 4). * *P* < 0.05, ** *P* < 0.01, *** *P* < 0.001 compared with no soluble tannin hydrolysate (0 μg/ml).

To further investigate the anti-inflammatory response of soluble tannin powder against MAC, we examined inflammatory cytokine gene expression and protein secretion by BMDMs (Figs 5 and 6). During MAC infection, gene expression of IL-1ß, IL-6, TNF-α and iNOS by BMDMs was significantly lower when treated with 100 μg/ml soluble tannin hydrolysate compared with control (0 μg/ml) (Fig 5). Moreover, protein levels of IL-1ß, IL-6, and TNF-α secreted by BMDMs treated with soluble tannin hydrolysate also decreased compared with the control (Fig 6).

## Discussion

Persimmon tannin in our study is a condensed tannin composed of a polymer of flavan-3-ols such as catechin groups [24–25]. In Japan, persimmon tannin is processed into kaki-shibu, and used for waterproofing and for dyeing leather and cloth. Recently, persimmon tannin was also used for the prevention of dental caries and biofilm formation [26–27]. We therefore hypothesized that soluble tannin hydrolysate could have antimicrobial activity against MAC, and it might thus prevent opportunistic MAC disease. Interestingly, soluble tannin powder had no significant effect against MAC *in vitro*, which suggests that it is necessary to hydrolyze soluble tannin into small molecules for its anti-bacteriostatic activity. In a previous study, we investigated the plasma ORAC value of animals fed a tannin-containing diet, and demonstrated that the plasma ORAC value of animals fed tannin was higher than the control diet group [15]. To increase plasma ORAC values, decomposition and absorption of tannin are required. Tannin was initially presumed to be nondigestible and unabsorbed, however, we hypothesized that tannin might be absorbed in the large intestine of animals after intestinal bacterial degradation. Notably, soluble tannin hydrolysate showed significantly higher ORAC values than soluble tannin *in vitro*. This suggests that hydrolysis of soluble tannin into small molecules is necessary for its bacteriostatic and antioxidant activity *in vivo*. Thus there might be a gap both in the degree of hydrolyzation and in the concentration of tannin between *in vivo* and *in vitro* studies.

Here we demonstrated in an *in vivo* animal model that the MAC bacterial count of lungs isolated from mice fed soluble tannin powder was significantly less than the control diet group. We also demonstrated formation of significantly smaller pulmonary granulomas in MAC-infected mice treated with the soluble tannin diet compared with the control diet group. Macrophages are the dominant cell type in granulomas, and can be activated by either classical (M1) or alternative (M2) pathways depending on the stimulus [8]. M1 macrophages, induced by proinflammatory molecules, play an important role in the elimination of various pathogens including mycobacteria such as MAC. They upregulate the expression of proinflammatory cytokines (IL-1ß, IL-6, TNF-α) and *via* activation of iNOS are able to secrete nitric oxide (NO), a free radical that is toxic to bacteria and other pathogens [28]. In granuloma macrophages, iNOS expression is induced following mycobacterial-induced granuloma formation [29]. Interestingly, hydrolysable tannins reduced iNOS protein expression by the murine macrophage-like cell line RAW264.7 [17] and murine macrophages [30]. Therefore, it is hypothesized that soluble tannin powder, which is a non-digestible macromolecule, was hydrolyzed into small molecules in vivo prior to performing its bacteriostatic activity and reducing granuloma size in lungs.

To confirm the anti-inflammatory effect of persimmon-derived tannin, we also evaluated expression of proinflammatory cytokines (IL-1ß, IL-6, TNF-α) and iNOS levels following MAC infection of BMDMs. Levels of proinflammatory cytokines and iNOS were significantly reduced by treatment with soluble tannin hydrolysate, which might help to regulate both excessive innate inflammatory and acquired Th1 immune responses. However, the downregulation of inflammatory cytokines by persimmon- derived tannin was less in the MAC disease animal model. This discrepancy in antioxidant activity between *in vitro* and *in vivo* results may be due to differences in the molecular size of hydrolyzed tannin. For instance, non-digestible macromolecules such as condensed tannin must become small molecules to exhibit their antioxidant activity *in vivo*. However, the mechanism regulating this was not proven in detail in this article. Chen et al. demonstrated that proanthocyanidins, another name for condensed tannins, ameliorate allergic contact dermatitis, directly inhibiting T cell activation and Th1/Th17 responses [31]. Thus there seem to be some similarities in the biological properties of condensed tannins. However, the optimal method of administration, concentration of persimmon fruit extract including tannin, and a detailed analysis of the chemical structure of soluble tannin (hydrolysate) should be determined in future research.

In this article, we elucidated that the soluble tannin hydrolysate has immunological benefits in MAC disease. Persimmon-derived tannin thus has potential medical applications and may be a valuable therapeutic candidate against opportunistic MAC infection.
